# Efficient Synthesis of Kinsenoside and Goodyeroside A by a Chemo-Enzymatic Approach

**DOI:** 10.3390/molecules191016950

**Published:** 2014-10-22

**Authors:** Yang Zhang, Yihong Xia, Yongji Lai, Fang Tang, Zengwei Luo, Yongbo Xue, Guangmin Yao, Yonghui Zhang, Jinwen Zhang

**Affiliations:** 1 Tongji Hospital Affiliated to Tongji Medical College, Huazhong University of Science and Technology, Wuhan 430030, China; E-Mail: nagexiaoshui@163.com; 2 Hubei Key Laboratory of Natural Medicinal Chemistry and Resource Evaluation, School of Pharmacy, Tongji Medical College, Huazhong University of Science and Technology, Wuhan 430030, China; E-Mails: zyhust123@163.com (Y.Z.); missxia55@163.com (Y.X.); laiyongji1024@163.com (Y.L.); luozengwei@hust.edu.cn (Z.L.); bobbyschro@126.com (Y.X.); gyap@mail.hust.edu.cn (G.Y.); zhangyh@mails.tjmu.edu.cn (Y.Z.)

**Keywords:** kinsenoside, goodyeroside A, chemo-enzymatic synthesis, glucosidase, biocatalysis

## Abstract

Kinsenoside (**1**) and goodyeroside A (**2**), two naturally occurring stereoisomers with diverse biological activities, have been synthesized efficiently by a chemo-enzymatic approach with a total yield of 12.7%. The aglycones, (*R*)- and (*S*)-3-hydroxy-γ-butyrolactone, were prepared from d- and l-malic acid by a four-step chemical approach with a yield of 75%, respectively. These butyrolactones were then successfully glycosidated using β-d-glucosidase as a catalyst in a homogeneous organic-water system. Under the optimized enzymatic conditions, the yields of kinsenoside and goodyeroside A in the enzymatic steps both reached 16.8%.

## 1. Introduction

**Figure 1:**
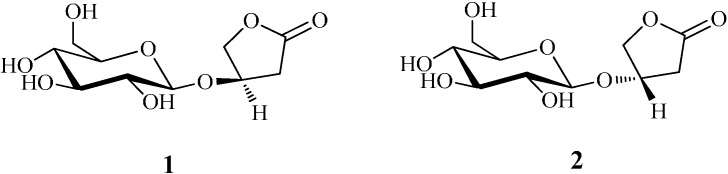
Structures of compounds **1** and **2**.

3-(*R*)-3-β-d-Glucopyranosyloxybutanolide (kinsenoside, **1**, Figure 1) has been isolated from three *Anoectochilus* species (Orchidaceae), namely, *A. koshunensis* [1], *A*. *formosanus* [2], and *A. roxburghii* [3]. 3-(*S*)-3-β-d-Glucopyranosyloxybutanolide (gooderoside A, **2**, Figure 1), the epimer of kinsenoside, was first isolated from *Crocus sativus* (Iridaceae) [4], and later from three *Goodyera* species (Orchidaceae) [5]. Preliminary *in vitro* pharmacological studies indicated that both compounds **1** and **2** had protective effects on primary cultured rat liver cells suffering from carbon tetrachloride exposure [5,6]. Moreover, pharmacological investigations on rats demonstrated that **1** possessed several other significant effects, including antihyperliposis [7], anti-inflammatory [8], antihyperglycemic [3], vascular protection [9], ovariectomy-induced bone loss preventive, and osteoclastogenesis suppressing properties [10]. In light of their effective physiological activities *in vitro* and *in vivo*, preparations of compounds **1** and **2** to meet the need of these compounds for further pharmacological studies have been described in the literature. In one of these approaches, the key chiral intermediates (*R*)- and (*S*)-3-hydroxy-γ-butyrolactone were initially synthesized, and then reacted with 2,3,4,6-tetra-*O*-benzyl-β-d-glucopyranosyl trichloroacetimidate to afford **1** and **2** in a total yield of 0.8% after seven steps [11]. In another study, optically inactive 1,2,4-butanetriol and 2,3,4,6-tetra-*O*-benzoyl-*α*-d-glucopyranosyl trichloroacetimidate as protected glucose were used to prepare **1** and **2** with a total yield of 10.7% over nine steps [12]. However, both these routes need protection and deprotection steps in the glycosylation process and have relatively circuitous synthetic routes. On the other hand, it is difficult to separate stereoisomeric products. In order to overcome these challenges, we have adopted a chemical process to synthesize 3-hydroxy-γ-butyrolactones from malic acid in three steps according to a reference procedure with some modifications. The obtained (*R*)- and (*S*)-3-hydroxy-γ-butyrolactones were reacted with β-d-glucose as glycosylation agent and β-d-glycosidase as catalyst to prepare kinsenoside (**1**) and goodyeroside A (**2**) separately. At the same time, reaction conditions for the enzymatic step were optimized by single factor and orthogonal experimental design.

## 2. Results and Discussion

Kinsenoside and goodyeroside A have been prepared efficiently by a five-step chemo-enzymatic route with a total yield of 12.7%, while the yield of a chemical approach to the 3-hydroxy-γ-butyrolactone intermediates was improved to 75%. Reaction conditions for the subsequent enzymatic step were optimized.

### 2.1. Improvement of the Chemical Approach

**Scheme 1:**
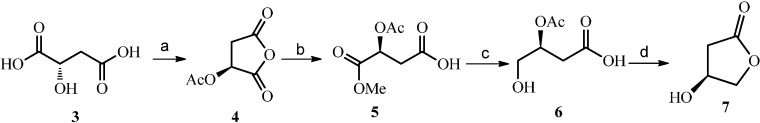
Chemical synthesis of compound **6** from compound **3**.

The synthesis of 3-hydroxy-γ-butyrolactone was significantly improved compared to previous methods (Scheme 1). Firstly, for post-processing of anhydride **4**, the acetic acid formed in the reaction was removed via the 1,4-dioxane-acetic acid azeotrope. Moreover, the duration of the reaction for preparing monomethyl ester **5** was remarkably reduced to 30 minutes, far less than the previously reported 12 h [13,14]. Additionally, in the last synthetic step, the reagent mixture composed of HCl-MeOH was replaced by HCl -H_2_O-1,4-dioxane, which significantly enhanced the purity of the ultimate products and simplified the separation step [13,14]. Under our optimized conditions, the yield of 75% was as high as described in prior references [13,14].

*Reagents and conditions*: a: AcCl, 40 °C, 4 h; b: MeOH, r.t., 30 min; c: NaBH_4_, i-BuOH, 85 °C, 2 h; d: HCl, 1.4-dioxane, 70 °C.

### 2.2. Construction and Optimization of Enzymatic Synthesis

**Scheme 2:**
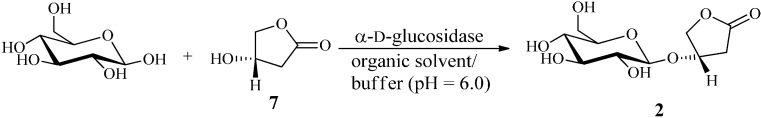
Enzymatic synthesis of compound **2**.

(*R*)- and (*S*)-3-Hydroxy-γ-butyrolactones, respectively, were successfully converted to their glucosides in a homogeneous organic-water medium (Scheme 2). Reaction conditions for the enzymatic step were optimized by single factor and orthogonal experimental design.

#### 2.2.1. Single Factor Experiments

**Figure 2:**
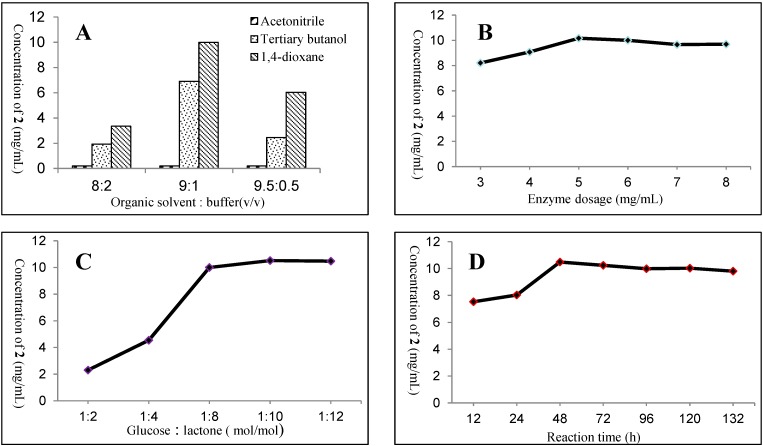
(**A**) Effects of different organic solvents and contents on the synthesis of **2** catalyzed by almond β-d-glycosidase in homogeneous systems; (**B**) effects of almond β-d-glycosidase dosages on the synthesis of **2**; (**C**) effects of (*S*)-3-hydroxy-γ-butyrolactone concentrations on the synthesis of **2**; (**D**) Dependence of **2** formation on reaction time. For single factor experiments, when one factor was investigated, the best condition of the three other factors was chosen.

In the initial study, three organic solvents and their concentrations at three levels (80%, 90% and 95%, *v*/*v*) were investigated for the enzymatic synthesis. As shown in Figure 2A, 1,4-dioxane was consistently the best solvent in comparison with *tert*-butanol and acetonitrile, regardless of concentrations. In particular, the solvent system consisting of 90% 1,4-dioxane and 10% buffer (*v*/*v*) displayed the best results in promoting the reaction result. Surprisingly, our results clearly demonstrated that acetonitrile was not applicable as a co-solvent for enzymatic glycosidation, regardless of buffer ratios. These results are in contrast with those of related studies [15,16], in which 90% *tert*-butanol or acetonitrile gave better results than 1,4-dioxane.

In order to further optimize the reaction and then use less amount of β-d-glucosidase, a series of reaction systems containing a range of enzyme amounts were tentatively established. As shown in Figure 2B, the process with the enzyme at a dose of 5 mg was favorable to accumulate **2**. No increased amount of glucoside was observed with more enzyme, but the amount of glucoside was reduced with the decreased enzyme dosage. Thus the dose of 5 mg glucosidase was chosen in this study.

Because enzymatic glycosidation is a thermodynamically-controlled reversable hydrolysis process, high concentrations of glucose and (*S*)-3-hydroxy-γ-butyrolactone were expected to give more glucoside. The maximum glucose concentration was 0.25 M in our chosen reaction medium consisting of 1,4-dioxane and buffer (9:1, *v*/*v*) [15]. The effect of the concentration of (*S*)-3-hydroxy-γ-butyrolactone on the yield of compound 2 was investigated, and the results showed that in the presence of 0.25 M glucose, the yield of **2** increased with the content of lactone and reached its maximum at a ratio of 1:10 (glucose: lactone) (Figure 2C). This ratio was finally chosen as the best condition in our study. In our organic-water medium, the reverse hydrolysis needed more time to reach thermodynamic equilibrium. As shown in Figure 2D, after 48 h, the reaction reached equilibrium and the concentration of products were also found to be 10.5 mg/mL. When the reaction duration was extended excessively, some glucoside might be converted back to glucose and 3-hydroxy-γ-butyrolactone.

#### 2.2.2. Orthogonal Experiments

As mentioned above, the reverse of the glucoside-producing reaction needed a certain amount of glucosidase and aglycone, as well as a defined duration to reach equilibrium. In order to expound on the intrinsic relationships among them, three factors including β-d-glucosidase dosage, (*S*)-3-hydroxy-γ-butyrolactone concentration and reaction time at four levels were chosen to carry out an orthogonal experiment (L_16_). According to intuitive analysis of the orthogonal experiment results (Table 1), the amount of lactone was the most remarkable factor within a certain range, followed by reaction time and enzyme dosage. The best estimated conditions include 5 mg glucosidase, 10 equivalents of lactone in 1 mL reaction medium and a reaction duration between 44 and 68 h, consistent with the experimental group 8, in which the concentration of **2** was 11.05 mg/mL, and the yield reached 16.8%. In the same way, we conducted the orthogonal experiment to synthesize **1**. Results were similar to that of experiments of **2** (Table S1 in Supporting Information). The above results demonstrated that there was no stereoselectivity for (*S*)- and (*R*)-3-hydroxy-γ-butyrolactone in the enzymatic step.

**Table 1:** Orthogonal experiment to determine the optimal enzymatic reaction conditions of **2**.

Effect Factor	Enzyme Dosage (mg)	Lactone Equivalents	Reaction Time (h)	Concentration of 2 (mg/mL)	Yield (%)
1	4	7	32	6.0	9.1
2	4	8	44	8.3	12.6
3	4	9	56	9.4	14.2
4	4	10	68	9.2	13.9
5	5	7	44	8.4	12.7
6	5	8	32	7. 8	11.8
7	5	9	68	8.8	13.3
8	5	10	56	11.1	16.8
9	6	7	56	7.5	11.4
10	6	8	68	9.3	14.1
11	6	9	32	8.7	13.2
12	6	10	44	9.1	13.8
13	7	7	68	8.1	12.3
14	7	8	56	8.5	12.9
15	7	9	44	9.6	14.5
16	7	10	32	9.1	13.8
k1	8.217	7.507	7.876		
k2	8.990	8.478	8.822		
k3	8.641	9.097	9.115		
k4	8.841	9.608	8.877		
R	0.773	2.101	1.239		

ki (i = 1, 2, 3, 4): Mean concentration of **2** at case i level of corresponding factor; R: range, namely ki_max_–ki_min_.

## 3. Experimental Section

### 3.1. Materials

β-d-Glucosidase (EC 3.2.1.21, 2.31 U/mg) from almond was purchased from Sigma Co. (St. Louis, MO, USA), and used without any further treatment; Malic acids were purchased from Aladdin Chemistry Co. Ltd. (Shanghai, China); Chemical reagents were obtained from Sinopharm Chemical reagent Co. Ltd. (Shanghai, China).

### 3.2. NMR, MS and HPLC-ELSD Analysis

The NMR spectra of 3-hydroxy-γ-butyrolactone, kinsenoside and goodyeroside A, were recorded in CDCl_3_ and pyridine-d_5_, respectively, using a Bruker 400 MHz spectrometer (Bruker, Karlsruhe, Germany). The corresponding solvent signals δ_H_ 7.24**/**δ_C_ 77.23 (CDCl_3_) and δ_H_ 8.74/δ_C_ 150.35 (pyridine-d_5_) were used as references. The MS spectra were recorded on a Thermo LCQ DECA. XP^plus^ ESI-MS (Thermo Fisher, Palo Alto, CA, USA). HPLC analysis of the glucoside was carried out on a Hitachi HPLC L-2000 instrument (Hitachi, Tokyo, Japan) equipped with an Alltech ELSD 2000 detector (Alltech, Vienna, VA, USA). Compounds **1** and **2** were analyzed on a 4.6 mm × 250 mm Ultimate AQ-C_18_ column with 100% ultrapure water as the eluent at a flow-rate of 0.6 mL/min. The temperature of the detector’s drift tube was 115 °C and the flow rate of N_2_ was 3.0 L/min. Concentrations of **1** and **2** were calculated from peak areas by using a calibration curve.

### 3.3. Chemical Synthesis of 3-Hydroxy-γ-butyrolactone

(*S*)- and (*R*)-3-hydroxy-γ-butyrolactone were synthesized using d- and l-malic acid as starting materials, respectively (Scheme 1). To a 100 mL flask was added l-malic acid (**3**, 13.4 g, 0.1 mol) and redistilled acetyl chloride (60 mL). The mixture was stirred at 40 °C for 4 h. Excess solvent was evaporated with added 1,4-dioxane and white solid **4** (15.6 g, 0.0987 mol) was obtained from the residue via recrystallization with a chloroform and petroleum ether system (*v*/*v* = 1:1, CHCl_3_/PE). Compound **4** was then dissolved in redistilled methanol (120 mL) and stirred 30 min at room temperature. Excess methanol was evaporated to yield the crude product monomethyl ester **5** (18.6 g, 0.0979 mol). After refluxing of NaBH_4_ (9.5 g, 0.245 mol) in *t*-BuOH (125 mL) for 2 h, a mixture of *t*-BuOH (150 mL) and MeOH (10 mL) containing compound **5** (18.6 g) was added dropwise to the mixture and then reflux was continued for 2 h. A white solid **6** was obtained by rotary evaporation. A mixed solvent of HCl/H_2_O/1,4-dioxane (*v*/*v*/*v* = 55:165:290) was added dropwise to the white solid at 70 °C to prepare (*S*)-3-hydroxy-γ-butyrolactone (**7**). After evaporation of the solvents, the white residue was extracted with THF (200 mL). The organic layers were combined, dried over anhydrous Na_2_SO_4_ and concentrated under vacuum to yield the crude product, which was further purified by flash chromatography on silica gel using petroleum ether/ethyl acetate (4:1) as the eluent to afford (*S*)-3-hydroxy-γ-butyrolactone (7.67 g, 75%) as a colorless or yellowish oil. 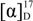 = +86.9 (*c* = 0.24, EtOH); 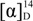 = +72.2 (*c* = 1.25, EtOH) in the reference [11]; ^1^H-NMR (CDCl_3_): δ_H_ 4.64–4.68 (m, 1H, H-3), 4.39 (dd, *J* = 10.3, 4.5 Hz, 1H, H-4b), 4.27 (d, *J* = 10.3 Hz, 1H, H-4a), 2.72 (dd, *J* = 18.0, 6.1 Hz, 1H, H-2b), 2.45–2.56 (m, 1H, H-2a). ^13^C-NMR (CDCl_3_): δ_C_ 37.8, 67.5, 76.0, 176.1.

The synthesis steps of (*R*)-3-hydroxy-γ-butyrolactone from D-malic acid were similar to those of (*S*)-3-hydroxy-γ-butyrolactone, with a yield of 75%. Colorless oil. 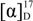 = −83.7 (*c* = 0.24, EtOH); 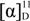 = −72.2 (*c* = 1.25, EtOH) in the reference [11]. ^1^H-NMR (CDCl_3_): δ_H_ 4.64–4.68 (m, 1H, H-3), 4.39 (dd, *J* = 10.3, 4.5 Hz, 1H), 4.24–4.29 (m, 1H, H-4a), 2.73 (dd, *J* = 18.0, 6.1 Hz, 1H), 2.50 (ddd, *J* = 18.0, 1.7, 1.1 Hz, 1H). ^13^C-NMR (CDCl_3_): δ_C_37.8, 67.5, 75.9, 176.1.

### 3.4. Enzymatic Synthesis of Compounds **1** and **2**

β-d-Glucosidase from almond (5 mg) was dissolved in phosphate buffer (Na_2_HPO_4_-KH_2_PO_4_, 70 mM, pH 6.0, 100 μL), then organic solvent (900 μL), 3-hydroxy-γ-butyrolactone (0.255 g, 2.5 mmol), and glucose (0.045 g, 0.25 mmol) were added to the reaction mixture. The reaction vessel was sealed and kept at 50 °C in thermostatic shaker with a shaking speed of 120 rpm for 48 h (Scheme 2). A water-bath set at 90 °C was used to quench the reaction. Concentrations of **1** and **2** were calculated from peak areas by using a calibration curve, and products were purified by silica gel column chromatography with chloroform/ethanol (4:1) as the eluent.

*Kinsenoside* (1): Colorless oil. 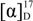 = +21.0 (*c* = 0.10, EtOH ); 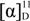 = +17.9 (*c* = 1.24, EtOH) in the reference [11]. ^1^H-NMR (pyridine-d_5_): δ_H_ 4.91 (d, *J* = 7.8 Hz, 1H, H-1'), 4.84–4.87 (m, 1H, H-3), 4.70 (d, *J* = 10.1 Hz, 1H, H-4a), 4.55 (d, *J* = 11.8 Hz, 1H, H-6'a), 4.34–4.45 (m, 2H, H-4b and H-6'b), 4.20–4.24 (m, 2H, H-4' and H-5'), 3.93–4.01 (m, 2H, H-3' and H-2'), 2.83–2.92 (m, 2H, H-2a and H-2b). ^13^C-NMR (pyridine-d_5_): δ_C_ 36.0, 63.0, 71.8, 75.1, 75.2, 75.6, 78.7, 79.1, 104.4, 176.4. ESI-MS: *m/z* 309.10 [M+HCOO]^−^ (Calcd for C_11_H_17_O_10_: 309.08).

*Goodyeroside A* (**2**): White crystals. mp 165–166 °C. 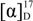 = −68.7 (*c* = 0.10, H_2_O); 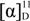 = −69.9 (*c* = 0.55, H_2_O) in the reference [11]. ^1^H-NMR (pyridine-d_5_) δ_H_ 4.94 (d, *J* = 7.7 Hz, 1H, H-1'), 4.87–4.91 (m, 1H, H-3), 4.66 (d, *J* = 10.1 Hz, 1H, H-4a), 4.55 (d, *J* = 11.8 Hz, 1H, H-6'a), 4.33–4.41 (m, 2H, H-4b and H-6'b), 4.19–4.26 (m, 2H, H-4' and H-5'), 3.92–4.01 (m, 2H, H-3' and H-2'), 2.86–2.95 (m, 2H, H-2a and H-2b). ^13^C-NMR (pyridine-d_5_): δ_C_36.8, 63.0, 71.8, 74.4, 75.0, 75.1, 78.7, 79.1, 104.0, 176.7. ESI-MS: *m/z* 309.10 [M+HCOO]^−^ (Calcd for C_11_H_17_O_10_: 309.08).

## 4. Conclusions

Kinsenoside (**1**) and goodyeroside A (**2**) were efficiently synthesized by a chemo-enzymatic approach with an overall yield of 12.7%. Aglycones were prepared by chemical approach with a yield of 75%. For the enzymatic approach, the yield of kinsenoside and goodyeroside A can reach to 16.8% under optimal reaction conditions, in which a 1 mL reaction system consisted of 1,4-dioxane (900 μL), phosphate buffer (Na_2_HPO_4_-KH_2_PO_4_, 70 mmol/L, pH 6.0, 100 μL), β-d-glucose (0.25 mmol), (*R*) or (*S*)-3-hydroxy-γ-butyrolactone (2.5 mmol) and β-d-glucosidase (5 mg); the reaction duration was 44–68 h, under 50 °C in thermostatic shaker with a shaking speed of 120 rpm.

## Supplementary Materials

Supplementary materials can be accessed at: http://www.mdpi.com/1420-3049/19/10/16950/s1.

## Supplementary Files

Supplementary File 1
