# Supplementary material for: Efficient Synthesis of Kinsenoside and Goodyeroside A by a Chemo-Enzymatic Approach
Source: Molecules. 2014 Oct 22;19(10):16950–8. doi: 10.3390/molecules191016950 (PMC6271766; doi:10.3390/molecules191016950)
Supplement: Supplementary File 1 [file molecules-19-16950-s001.pdf]

Supporting Information

Figure S1. ESIMS spectrum of **1**
Figure S2. ^{1}H-NMR spectrum (400 MHz) of **1** in C_5D_5N
Figure S3. ^{13}C-NMR spectrum (100 MHz) of **1** in C_5D_5N
Figure S4. HSQC spectrum of **1** in C_5D_5N
Figure S5. HMBC spectrum of **1** in C_5D_5N
Figure S6. HPLC analysis the purity of compound **1**
Figure S7. ESIMS spectrum of **2**
Figure S8. ^{1}H-NMR spectrum (400 MHz) of **2** in C_5D_5N
Figure S9. ^{13}C-NMR spectrum (100 MHz) of **2** in C_5D_5N
Figure S10. HSQC spectrum of **2** in C_5D_5N
Figure S11. HMBC spectrum of **2** in C_5D_5N
Figure S12. HPLC analysis the purity of compound **2**
Figure S13. ^{1}H-NMR spectrum (400 MHz) of **6** in $CDCl_3$
Figure S14. ^{13}C-NMR spectrum (100 MHz) of **6** in$CDCl_3$
Figure S15. ^{1}H-NMR spectrum (400 MHz) of (*R*)-3-hydroxy-*γ*-butyrolactone in $CDCl_3$
Figure S16. ^{13}C-NMR spectrum (100 MHz) of (*R*)-3-hydroxy-*γ*-butyrolactone in $CDCl_3$
Table S1. Orthogonal experiment to determine the optimal enzymatic reaction conditions of **1**.

Figure S1. ESIMS spectrum of **1**.

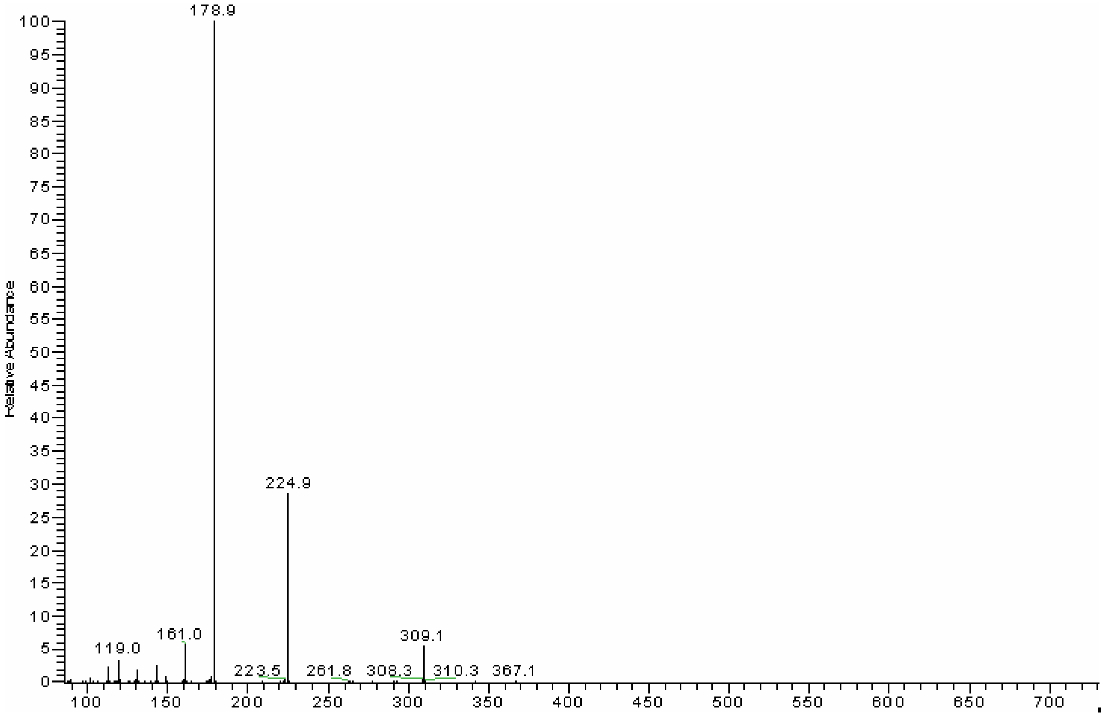

Figure S2. ^{1}H-NMR spectrum (400 MHz) of **1** in C_5D_5N.

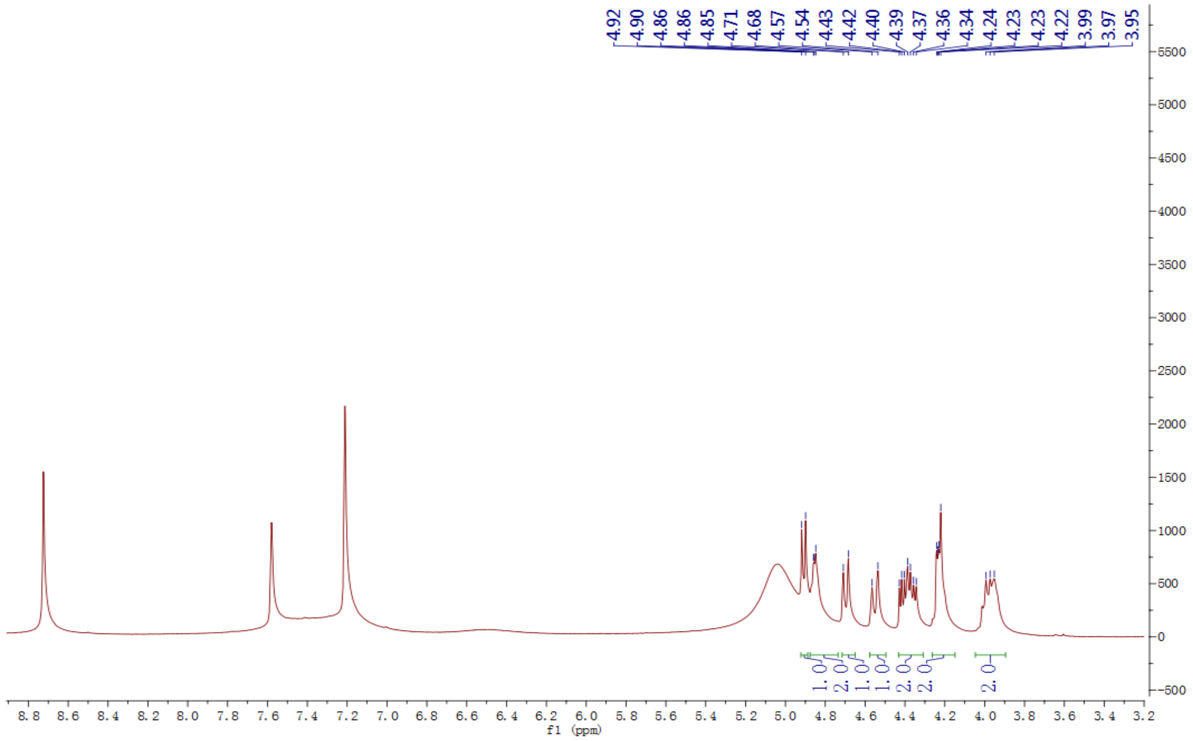

Figure S3. ^{13}C-NMR spectrum (100 MHz) of **1** in C_5D_5N.

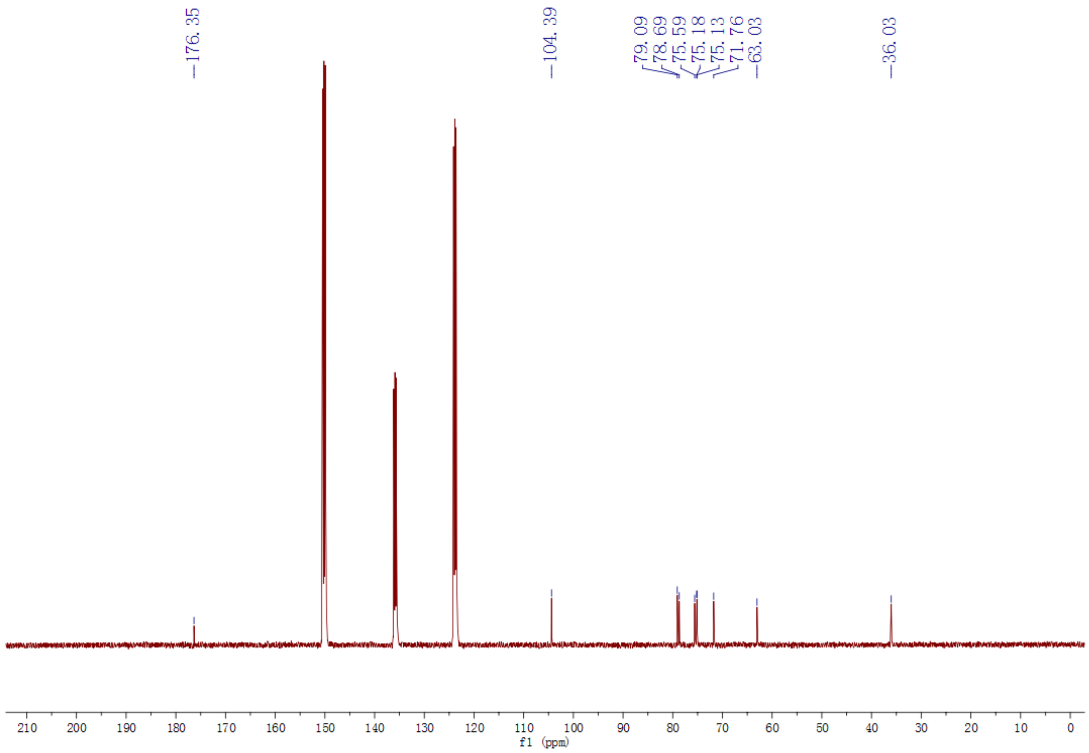

Figure S4. HSQC spectrum of **1** in C_5D_5N.

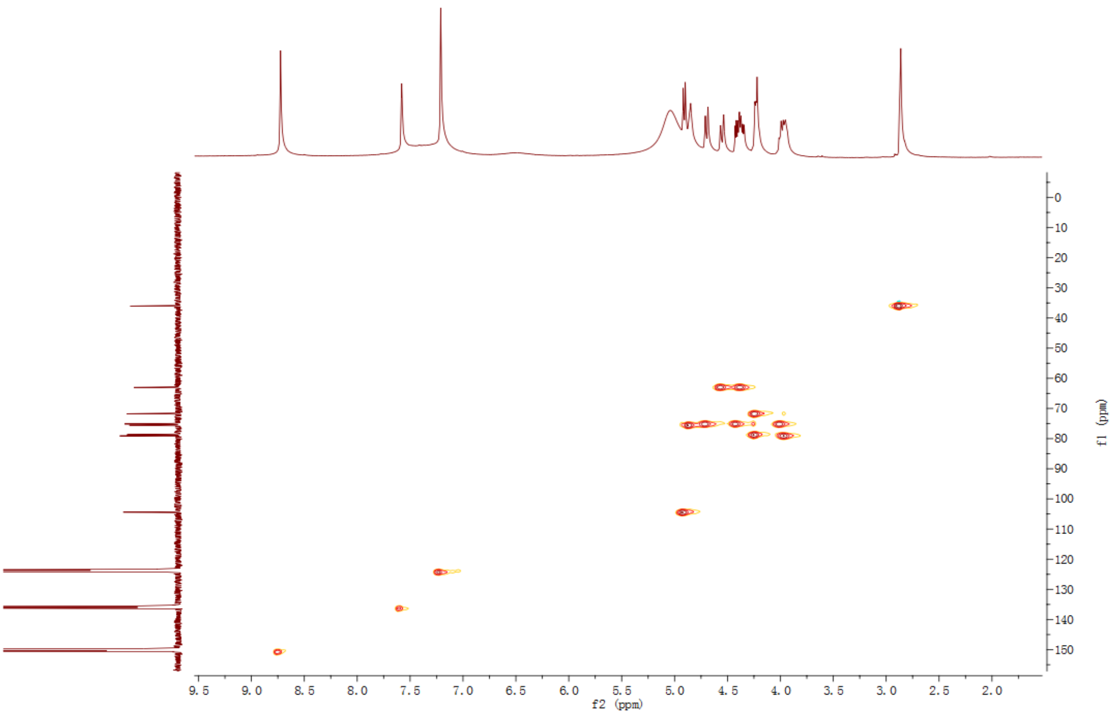

Figure S5. HMBC spectrum of **1** in C_5D_5N.

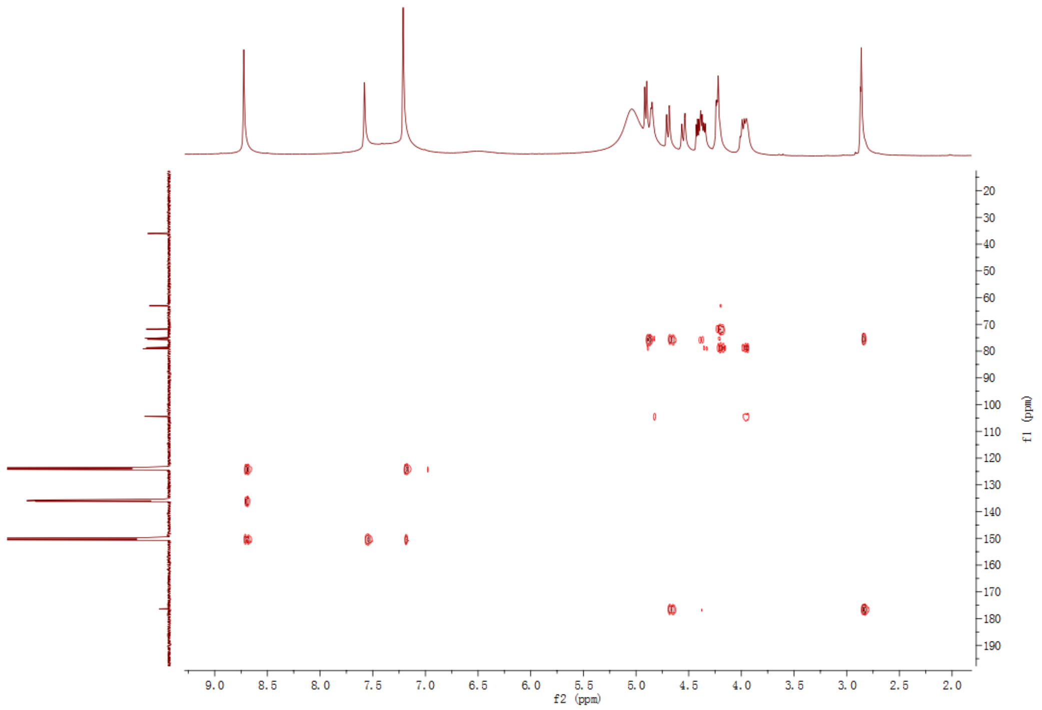

Figure S6. HPLC analysis the purity of compound **1**.

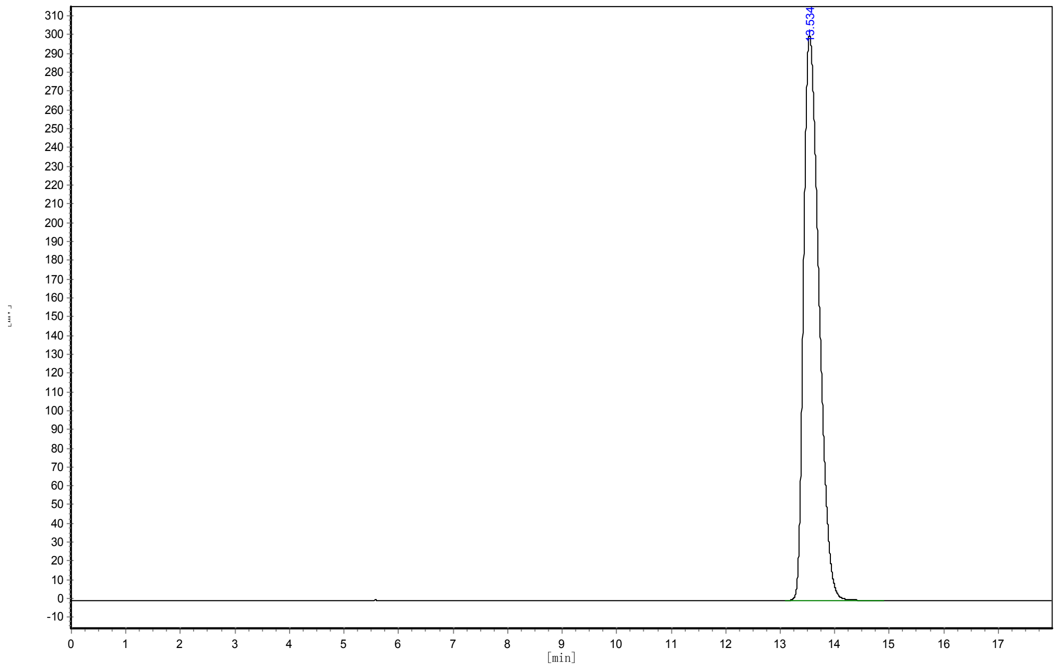

Figure S7. ESIMS spectrum of **2**.

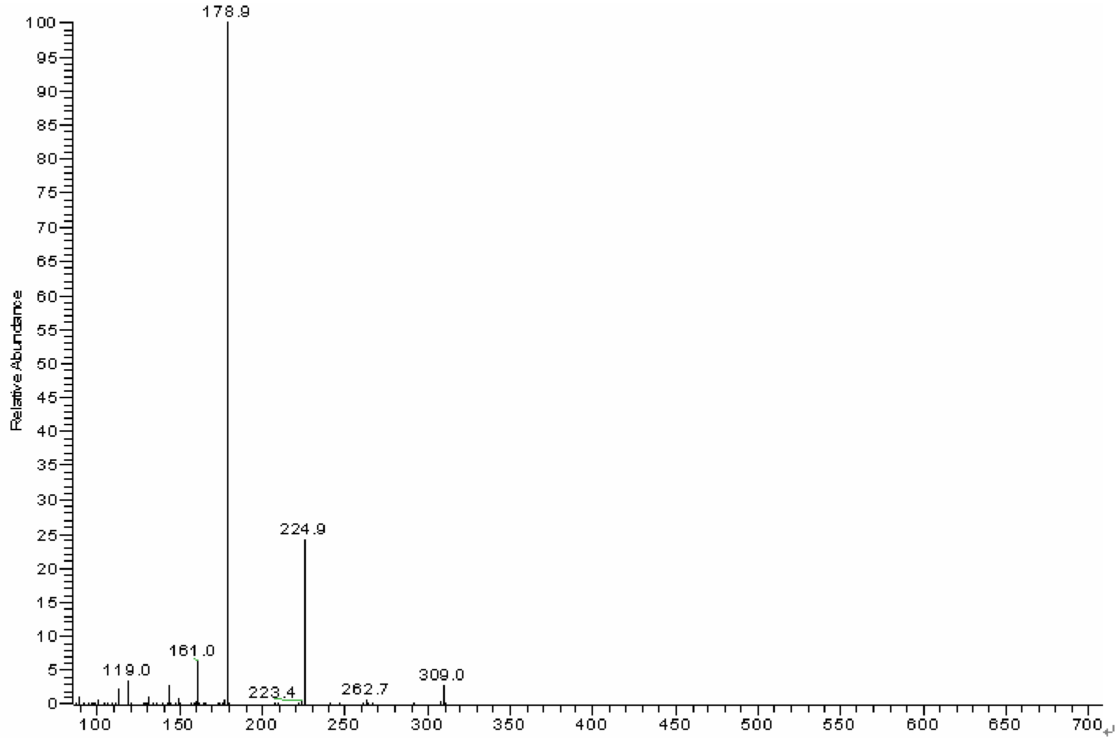

Figure S8. ^{1}H-NMR spectrum (400 MHz) of **2** in C_5D_5N.

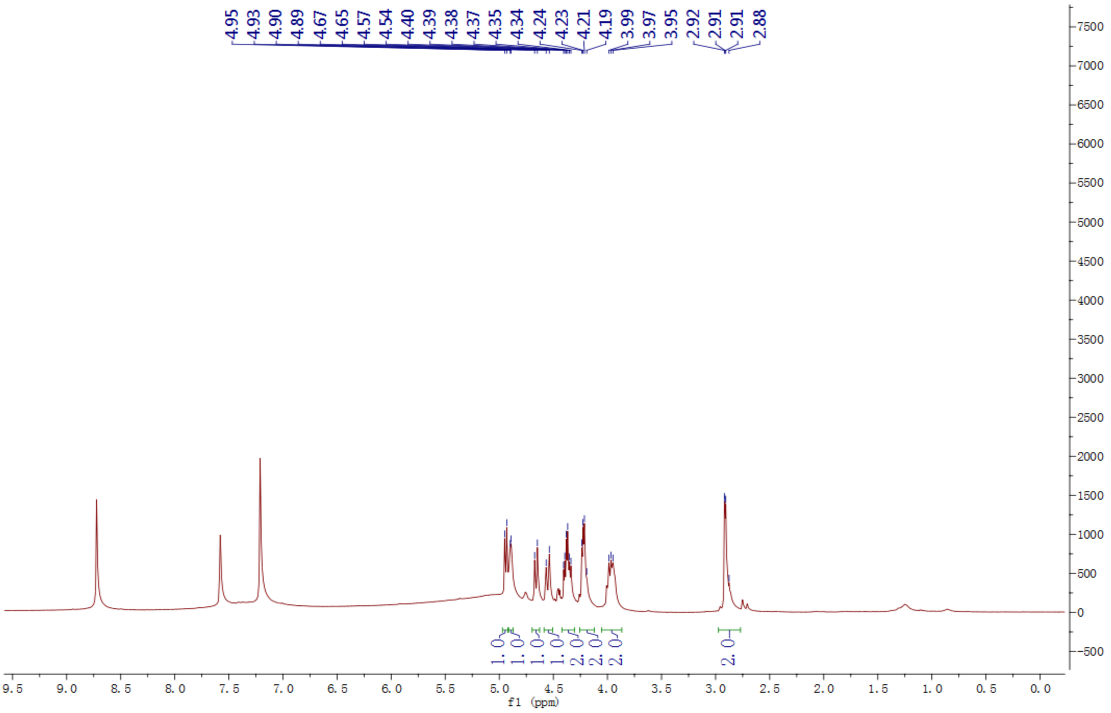

Figure S9. ^{13}C-NMR spectrum (100 MHz) of **2** in C_5D_5N.

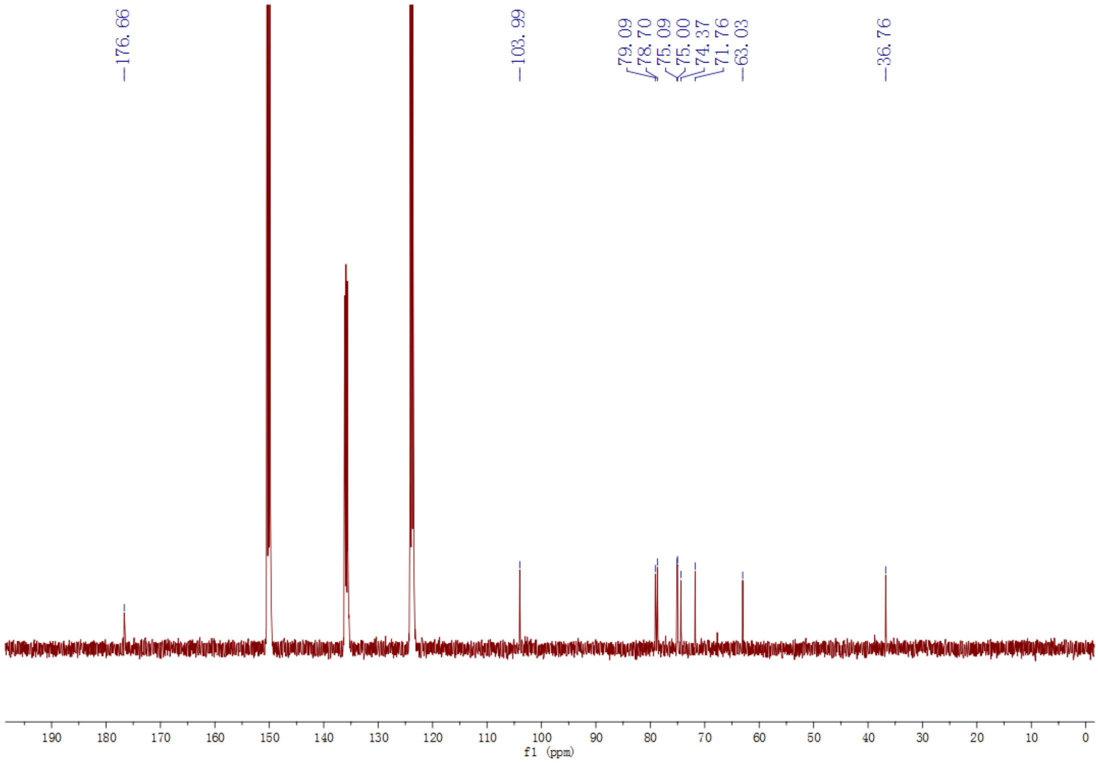

Figure S10. HSQC spectrum of **2** in $CDCl_3$.

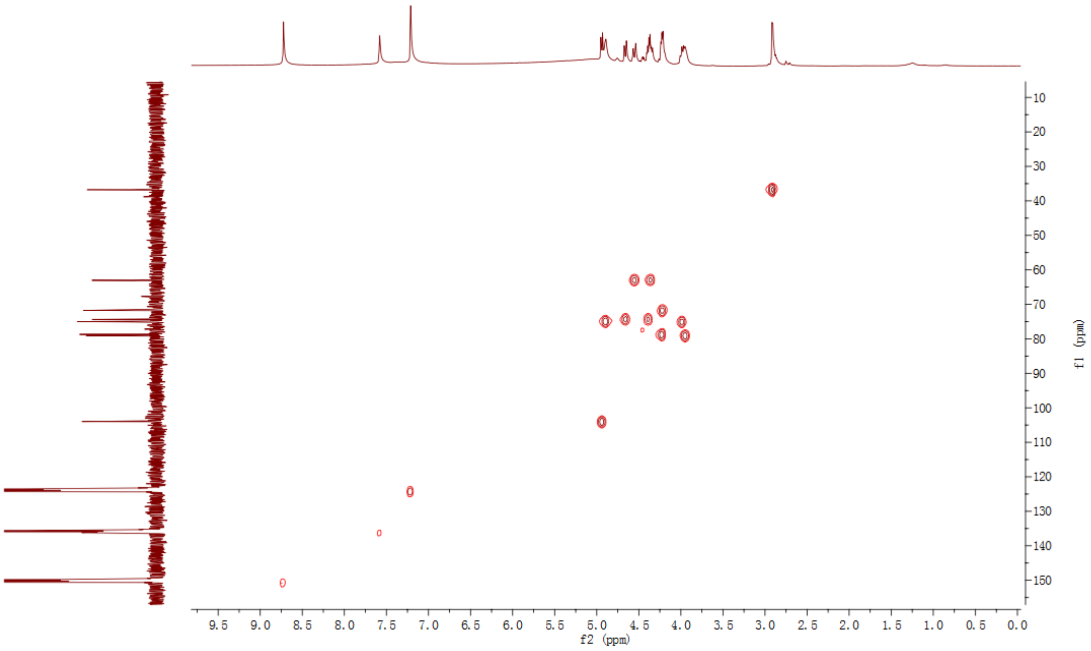

Figure S11. HMBC spectrum of **2** in C_5D_5N.

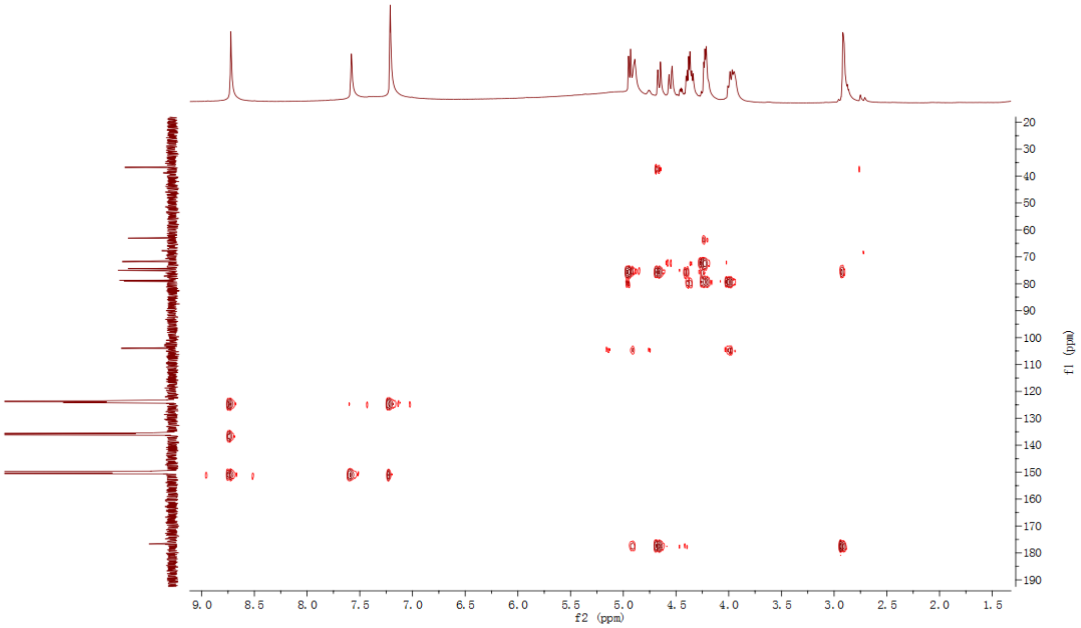

Figure S12. HPLC analysis the purity of **2**.

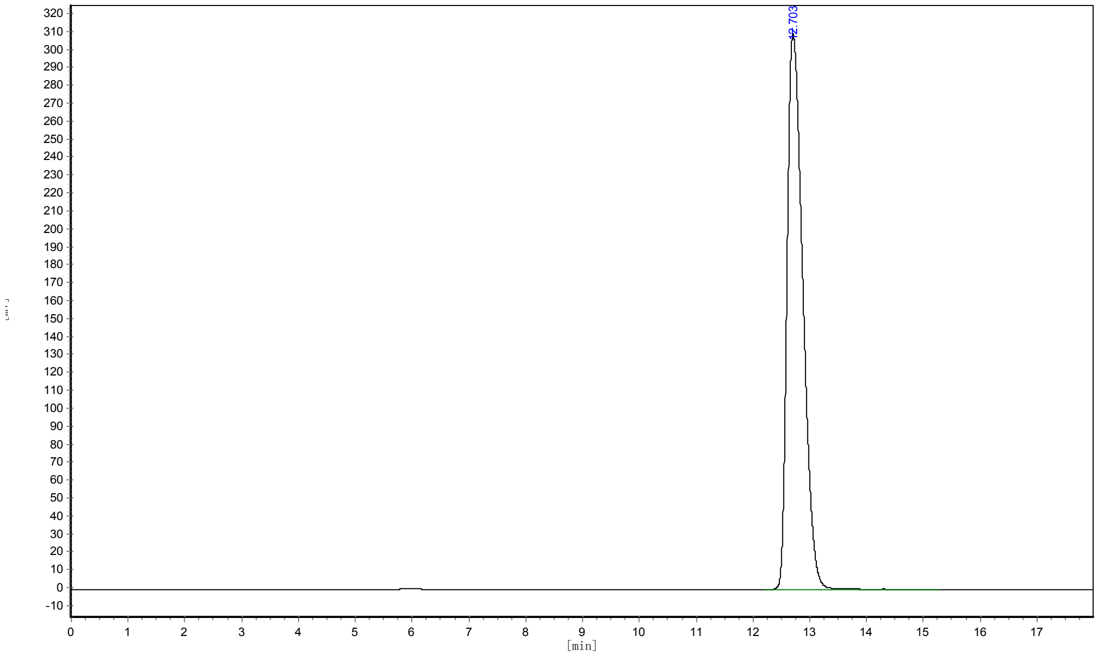

Figure S13. ^{1}H-NMR spectrum (400 MHz) of **6** in $CDCl_3$.

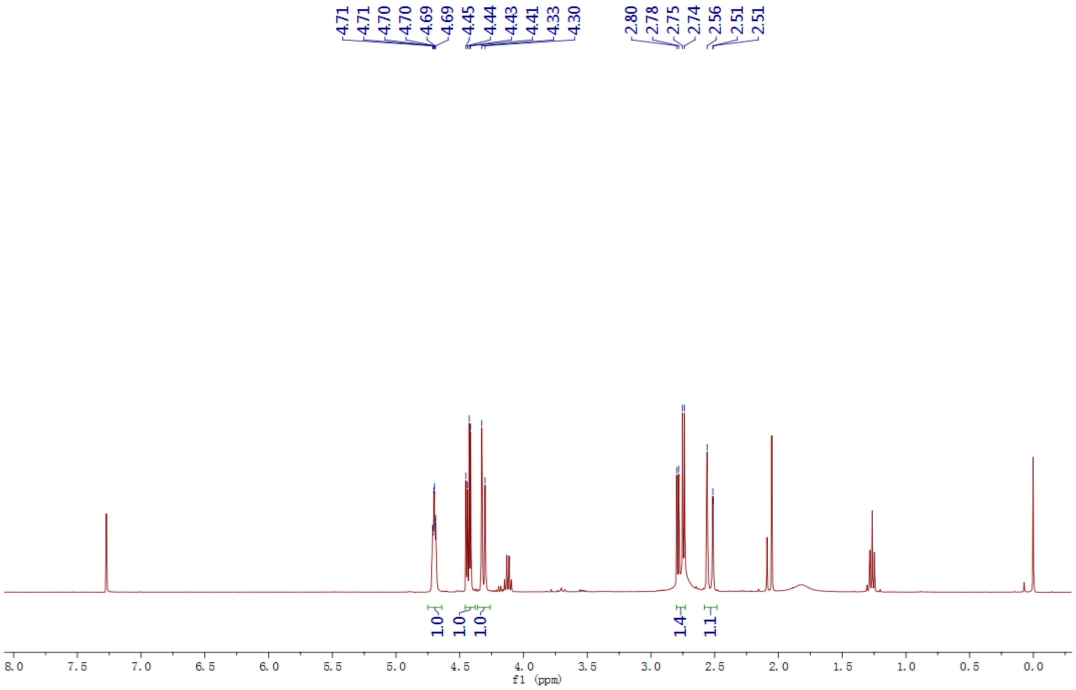

Figure S14. ^{13}C-NMR spectrum (100 MHz) of **6** in$CDCl_3$.

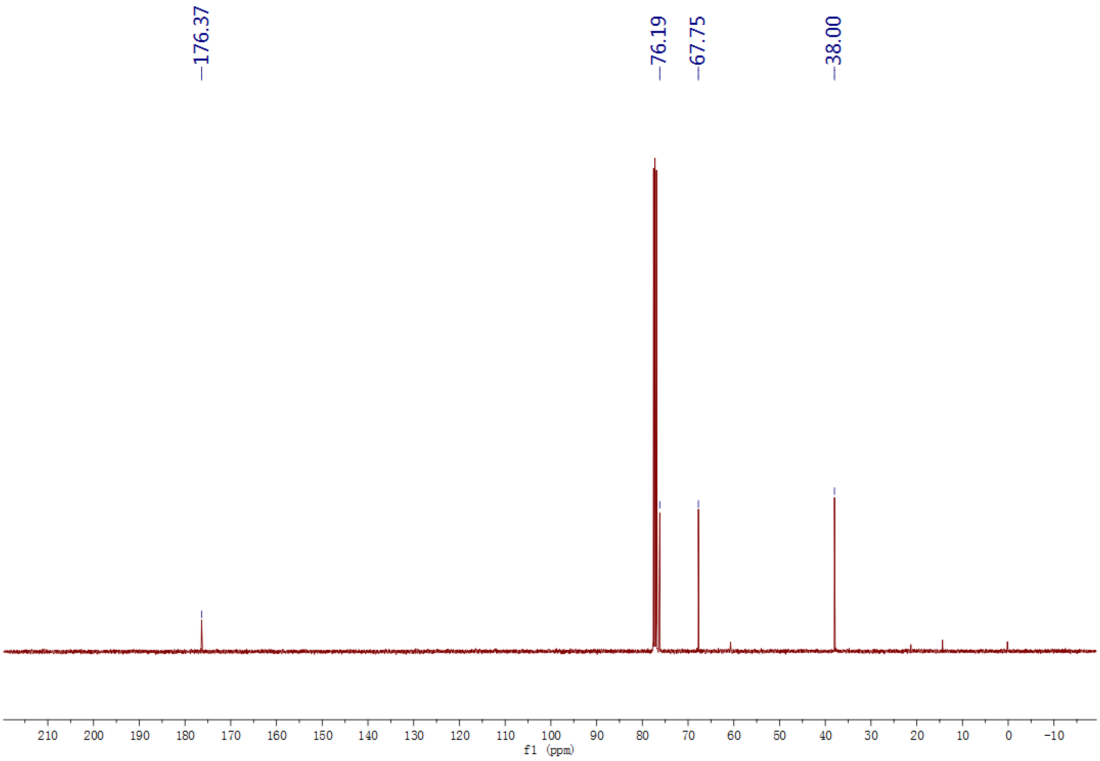

Figure S15. ^{1}H-NMR spectrum (400 MHz) of (*R*)-3-hydroxy-γ-butyrolactone in $CDCl_3$.

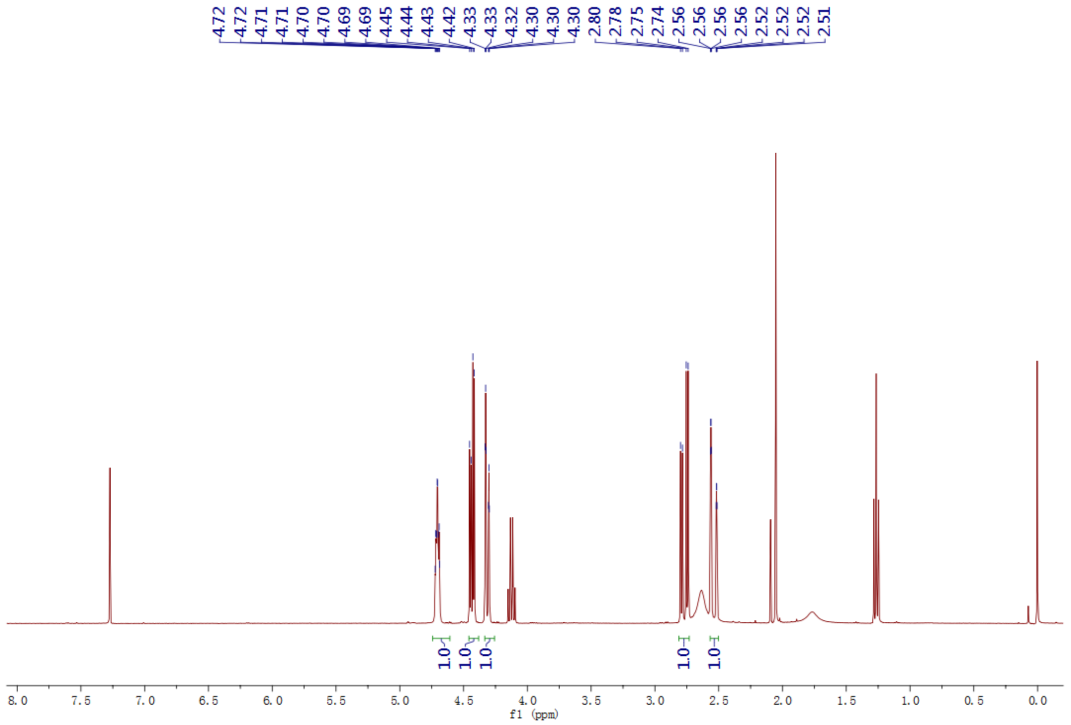

Figure S16. ^{13}C-NMR spectrum (100 MHz) of (*R*)-3-hydroxy-γ-butyrolactone in $CDCl_3$.

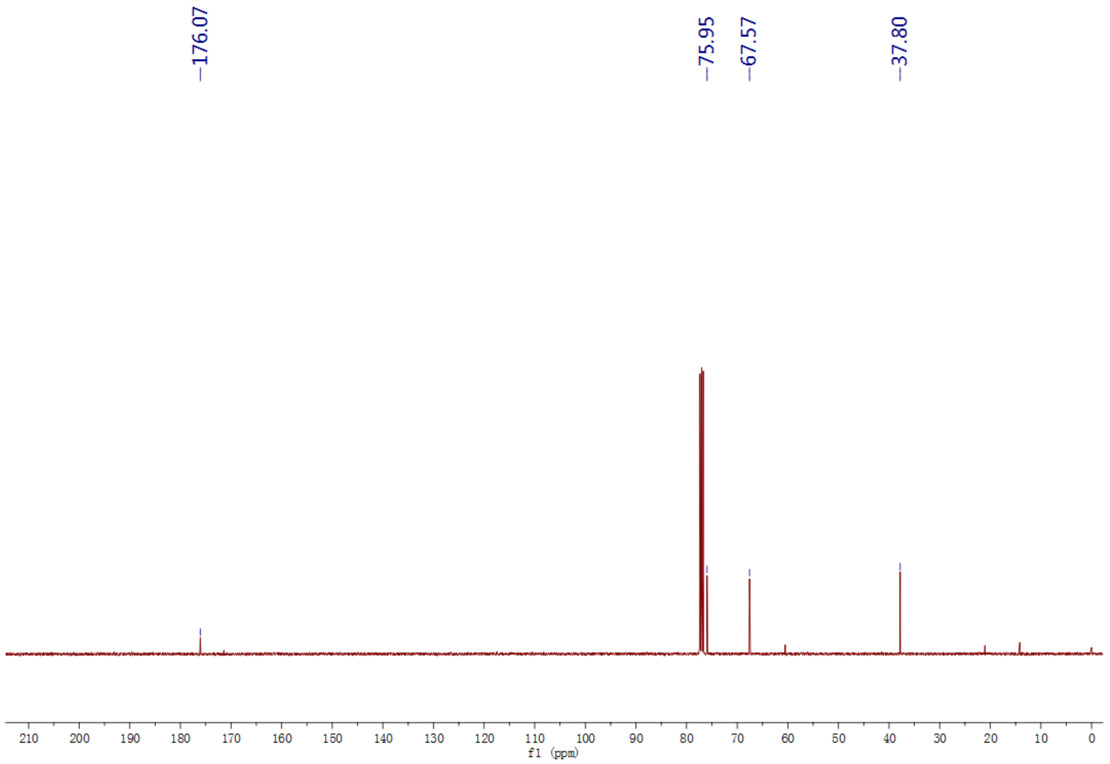

Table S1. Orthogonal experiment to determine the optimal enzymatic reaction conditions of **1**.

Effect Factor	Enzyme Dosage (mg)	Lactone Equivalents	Reaction Time (h)	Concentration of 1 (mg/mL)	Yield (%)
1	4	7	32	7.6	11.5
2	4	8	44	9.4	14.2
3	4	9	56	10.6	16.1
4	4	10	68	11.2	16.9
5	5	7	44	8.8	13.3
6	5	8	32	9.0	13.6
7	5	9	68	11.4	17.2
8	5	10	56	11.2	16.9
9	6	7	56	8.2	12.4
10	6	8	68	9.2	13.9
11	6	9	32	9.2	13.9
12	6	10	44	10.8	16.4
13	7	7	68	8.6	13.0
14	7	8	56	9.7	14.7
15	7	9	44	9.8	14.8
16	7	10	32	10.7	16.2
k1	9.723	8.315	9.165		
k2	10.119	9.338	9.717		
k3	9.376	10.262	9.923		
k4	9.701	11.003	8.877		
R	0.743	2.688	1.239		

ki (i = 1, 2, 3, 4): Mean concentration of **1** at case i level of corresponding factor; R: Range, namely ki_{max}–ki_{min}.
